# Loss of the Cyclin-Dependent Kinase Inhibitor 1 in the Context of Brachyury-Mediated Phenotypic Plasticity Drives Tumor Resistance to Immune Attack

**DOI:** 10.3389/fonc.2018.00143

**Published:** 2018-05-03

**Authors:** Duane H. Hamilton, Kristen K. McCampbell, Claudia Palena

**Affiliations:** Laboratory of Tumor Immunology and Biology, Center for Cancer Research, National Cancer Institute, National Institutes of Health, Bethesda, MD, United States

**Keywords:** epithelial–mesenchymal transition, phenotypic plasticity, brachyury, immune resistance, CDKN1A, CDK1

## Abstract

The acquisition of mesenchymal features by carcinoma cells is now recognized as a driver of metastasis and tumor resistance to a range of anticancer therapeutics, including chemotherapy, radiation, and certain small-molecule targeted therapies. With the recent successful implementation of immunotherapies for the treatment of various types of cancer, there is growing interest in understanding whether an immunological approach could be effective at eradicating carcinoma cells bearing mesenchymal features. Recent studies, however, demonstrated that carcinoma cells that have acquired mesenchymal features may also exhibit decreased susceptibility to lysis mediated by immune effector cells, including antigen-specific CD8^+^ T cells, innate natural killer (NK), and lymphokine-activated killer (LAK) cells. Here, we investigated the mechanism involved in the immune resistance of carcinoma cells that express very high levels of the transcription factor brachyury, a molecule previously shown to drive the acquisition of mesenchymal features by carcinoma cells. Our results demonstrate that very high levels of brachyury expression drive the loss of the cyclin-dependent kinase inhibitor 1 (p21CIP1, p21), an event that results in decreased tumor susceptibility to immune-mediated lysis. We show here that reconstitution of p21 expression markedly increases the lysis of brachyury-high tumor cells mediated by antigen-specific CD8^+^ T cells, NK, and LAK cells, TNF-related apoptosis-inducing ligand, and chemotherapy. Several reports have now demonstrated a role for p21 loss in cancer as an inducer of the epithelial–mesenchymal transition. The results from the present study situate p21 as a central player in many of the aspects of the phenomenon of brachyury-mediated mesenchymalization of carcinomas, including resistance to chemotherapy and immune-mediated cytotoxicity. We also demonstrate here that the defects in tumor cell death described in association with very high levels of brachyury could be alleviated *via* the use of a WEE1 inhibitor. Several vaccine platforms targeting brachyury have been developed and are undergoing clinical evaluation. These studies provide further rationale for the use of WEE1 inhibition in combination with brachyury-based immunotherapeutic approaches.

## Introduction

Studies from our laboratory and others have previously established the ability of the transcription factor brachyury (gene name *T*), a member of the T-box family, to promote the acquisition of mesenchymal features by carcinoma cells (1–5). Gain- and loss-of-function experiments have shown that brachyury expression in epithelial cancer cells associates with (a) enhanced expression of mesenchymal proteins and reduction of epithelial proteins; (b) acquisition of tumor motility, invasiveness, and propensity to disseminate *in vivo* in xenograft models; (c) acquisition of stemness features, including a relatively quiescent state; and (d) conversion into a refractory, therapy-resistant state. The effects of brachyury on tumor phenotype were attributed to its ability to bind a half T-box DNA-binding site in the promoter of E-cadherin, in cooperation with the repressor Slug, resulting in decreased E-cadherin expression (1).

Brachyury has been found to be overexpressed in various human carcinomas, both in the primary tumor and metastatic sites, including in non-small cell (NSCLC) and small cell (SCLC) lung cancer (6, 7), triple-negative breast (TNBC) cancer (8, 9), prostate (4), and colon cancer (10, 11), among others. Interestingly, several reports have also shown the prognostic value of high brachyury expression at the primary tumor site, with high brachyury mRNA or protein levels being associated with poor clinical outcome, including in breast (8, 9), lung (12), colon (10), and prostate (4, 13).

There are several vaccine platforms targeting the transcription factor brachyury undergoing Phase I and II clinical evaluation (14–16). It has been recently shown, however, that the acquisition of mesenchymal features by carcinoma cells may decrease their susceptibility to lysis by immune effector cells, including antigen-specific CD8^+^ T cells, innate natural killer (NK), and lymphokine-activated killer (LAK) cells (17, 18). In the case of brachyury-mediated mesenchymalization of human carcinoma cells, the mechanism of immune resistance was identified as a defect in the phosphorylation and subsequent cleavage of the nuclear lamins during apoptosis, a defect caused by the loss of the cell-cycle dependent kinase-1 (CDK1), due to decreased protein stability (17). The reason for the decreased stability of the CDK1 protein in the presence of high levels of brachyury, however, was not previously elucidated. In the present study, we further investigated the mechanism involved in the immune resistance of carcinoma cells that express high levels of brachyury, and demonstrate that the loss of the cyclin-dependent kinase inhibitor 1 (p21CIP1, hereafter termed p21) is critical for the defective lysis of these cancer cells. Reconstitution of p21 expression in brachyury-high tumor cells was shown to increase the stability of the CDK1 protein while markedly increasing the lysis mediated by antigen-specific CD8^+^ T cells, NK and LAK cells, TNF-related apoptosis-inducing ligand (TRAIL) and chemotherapy. The loss of p21 has been associated with the occurrence of epithelial–mesenchymal plasticity in cancer. The results from this work indicate that p21 plays a major role in many of the aspects of this phenomenon, including as an inducer of resistance to cell death in response to chemotherapy or immune effector cells.

## Materials and Methods

### Cell Culture

The following human carcinoma cell lines were obtained from American Type Culture Collection and propagated in recommended media: pancreatic PANC-1; lung H1299 and H460, colon HCT116. The murine MC38 colon adenocarcinoma cell line has been previously described (19). The full-length human brachyury and p21CIP1 encoding fragments were purchased from Origene (Rockville, MD, USA) and subsequently cloned into the pcDNA3.1(+) expression vector (Thermo Fisher Scientific). Tumor cells were stably transfected using a nucleofection device (Lonza) by following the manufacturer’s recommendations. For generation of H460 clones with various levels of brachyury, the GeneArt Precision gRNA synthesis kit (Invitrogen) was used for preparation of brachyury-specific gRNA designed *via* the use of the GeneArt CRISPR Search and Design online tool (Invitrogen). H460 cells were co-transfected with GeneArt Platinum Cas9 nuclease (Invitrogen) and brachyury-targeting gRNA by following the manufacturer’s instructions, and subsequently grown and seeded for single cell sub-culture onto 96-well plates. Clonally derived cell lines with various levels of brachyury were generated by using a limiting dilution cloning strategy. Clones were selected for further study based on the level of brachyury protein expression.

### Immune Effector Cells, Cytotoxicity Assays

Peripheral blood used in this study was obtained from healthy human donors recruited at the NIH Blood Bank (Bethesda, MD, USA), protocol number NCT00001846, under the appropriate NIH Institutional Review Board approval and informed consent. NK cells were isolated using human CD56 MicroBeads (Miltenyi Biotec). For generation of LAK cells, purified NK cells were incubated overnight in RPMI-1640 supplemented with 10% human AB sera and 2,000 U/mL of recombinant human IL-2 (Peprotech). T cells specific for the MUC1 HLA-A2 restricted peptide p93L (ALWGQDVTSV) were previously described (20). TRAIL-mediated lysis of tumor cells was performed by incubation with recombinant, active multimeric killer TRAIL (Enzo Life Sciences). The murine cytotoxic T-cell lines specific for p15E (KSPWFTTL) were expanded *in vitro* from splenocytes originating from either wild-type or perforin-deficient C57BL/6 mice, which had been vaccinated with the p15E peptide. Perforin-deficient animals were purchased from Jackson Laboratories (Bar Harbor, ME, USA). All animal studies were carried out in accordance with the guidelines of the Association for Assessment and Accreditation of Laboratory Animal Care (AAALAC). Experimental studies were carried out under approval of the NIH Intramural Animal Care and Use Committee. Where indicated, perforin/granzyme-mediated lysis of target cells was inhibited by incubating T cells with 200 nM Concanamycin A (CMA) (Sigma) for 2 h at 37°C prior to plating with target cells in the lysis assays. Target cells were labeled with 50 μCi ^111^In (GE Healthcare) and incubated overnight with TRAIL or effector NK, LAK, or T cells at indicated effector-to-target (E:T) ratios. Following overnight culture, supernatants were harvested for radioactivity assessment using a Wizard^2^ gamma counter (PerkinElmer). Specific lysis was calculated as previously described (21). Sensitivity of H460 cells to TRAIL lysis was performed using a luminescence-based viability assay. Cells were plated in white-walled 96-well trays and allowed to attach overnight. Using six-well replicates, cells were treated with indicated doses of recombinant Superkiller TRAIL (Enzo Life Sciences) and incubated for 16 h. Cell viability was assessed using CellTiter-Glo (Promega) according to the manufacturer’s instructions. Cell death was calculated as the percent reduction in luminescence in treated wells as compared to non-treated controls.

### Western Blot

Cells were washed twice with PBS and lysed in RIPA Lysis Buffer (Santa Cruz Biotech). Protein concentration was measured using a BCA Protein Assay Kit (Thermo Scientific). Aliquots containing 10–30 µg of protein were run on SDS-PAGE and transferred to nitrocellulose membranes. Following blockade for 1 h at room temperature with 5% milk in PBS, the membranes were probed overnight at 4°C using antibodies specific for pan-actin (clone Ab-5, Neo Markers), GAPDH (Santa Cruz Biotechnology), p21 (clone 12D1, Cell Signaling Technology), vimentin (clone RV202, BD Biosciences), E-cadherin (clone 36/E-cadherin, BD Biosciences), CDK1 (Cell Signaling Technology), and brachyury MAb 54-1 (22). CDK1 immunoprecipitation was performed using 200 µg of cleared cell extract using either a control rabbit IgG (Abcam) or anti-CDK1 antibody (Upstate). Antibody/protein complexes were purified using Protein G beads (Sigma), and the eluted protein was assessed for the presence of CDK1 and p21 by western blot. Protein synthesis was inhibited by incubating tumor cells with 100 µg/mL cycloheximide (Sigma); cells were harvested at various time points and CDK1 protein levels were assessed by western blot. All blots were imaged using the Odyssey Infrared imaging system (LI-COR Biotechnology).

### Real-Time PCR

Total RNA was isolated from frozen cell pellets using the RNeasy kit (QIAGEN), and cDNA was reverse transcribed with Advantage RT-for-PCR (Clontech). cDNA (1–100 ng) was amplified in triplicate using Gene Expression Master Mix and the following TaqMan gene expression assays (Thermo Fisher Scientific): brachyury (Hs00610080), and GAPDH (4326317E). Mean Ct values for target genes were normalized to mean Ct values for the endogenous control GAPDH [−ΔCt = Ct(GAPDH) − Ct(target gene)]. The ratio of mRNA expression of target gene versus GAPDH was defined as 2(−ΔCt).

### MK-1775 Treatments

The WEE1-inhibitor, MK-1775, utilized in these studies was purchased from Selleckchem. Carcinoma cell lines were treated with indicated concentrations of MK-1775 for 72 h prior to the addition of immune effector cells or a combination of cisplatin (APP Pharmaceuticals) and vinorelbine (Bedford Laboratories). To evaluate responses to chemotherapy, tumor cells were incubated for 4 h in the presence of the chemotherapeutic agents; MK-1775 was present for the entirety of the assay. Cell viability was assessed 3–4 days after exposure to chemotherapy using an MTT (Sigma) assay, as previously described (23).

### *In Silico* Analysis of The Cancer Genome Atlas (TCGA) Dataset

Relative expression levels of indicated mRNAs were assessed using the TCGA dataset containing data from 482 lung squamous cell carcinoma patients (24). For the analysis, samples were subdivided into four groups according to the level of brachyury (T) expression: 318 of 482 samples with no detectable brachyury expression were classified as negative (neg.). The remaining 164 samples were ranked and subdivided into tertiles based upon the level of brachyury expression: “brachyury-high” (55 of 482), “brachyury-intermediate” (55 of 482), and “brachyury-low” (54 of 482) groups. Samples in the intermediate and low groups were combined; the level of expression of mRNA encoding E-cadherin (*CDH1*), p21 (*CDKN1A*), and CDK1 (*CDK1*) were evaluated in each group. All data were analyzed using the Nexus Expression 3 analysis software package (BioDiscovery).

### Statistical Analysis

Unless indicated all statistical comparisons between two sample groups were performed using a two-tailed unpaired Student’s *t*-test on triplet technical replicates using Prism 7 (GraphPad).

## Results

### High Expression of Brachyury Induces Resistance to TRAIL Lysis

**Figure 1:**
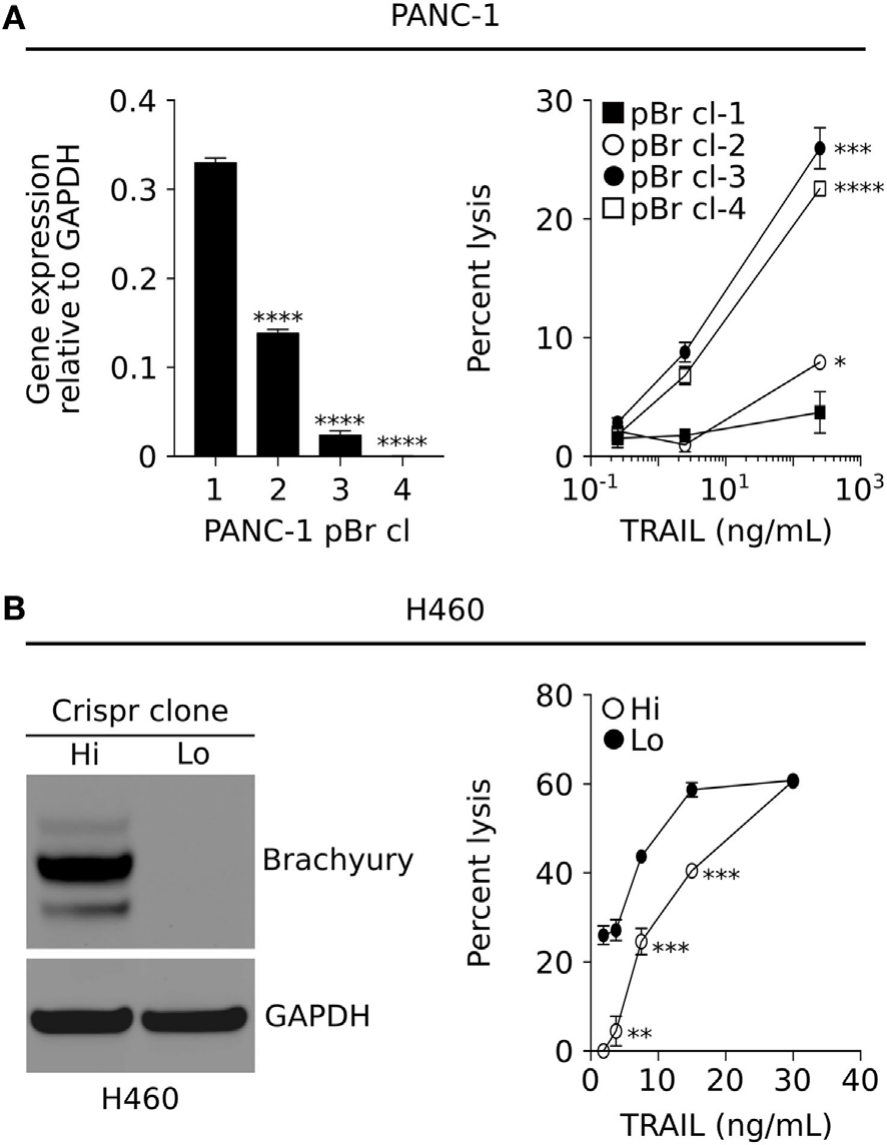
Brachyury associates with increased resistance to immune-mediated killing. **(A)** Relative brachyury expression in four clonally derived human PANC-1 cell lines (left panel), and their associated sensitivity to lysis by TNF-related apoptosis-inducing ligand (TRAIL) (right panel). **(B)** Western blot analysis of brachyury expression in two clonally derived populations of H460 generated *via* the CRISPR approach (left panel), and their sensitivity to lysis by TRAIL (right panel). Presented data are representative of three independent experiments.

To better understand the role of brachyury in tumor resistance mechanisms, we utilized various models of clonally derived tumor cell populations with different levels of brachyury. One such model was generated by stable transfection of the human pancreatic PANC-1 carcinoma cell line to overexpress human brachyury, followed by a clonal selection of single-cells with a range of brachyury expression (Figure 1A, left panel). As shown in Figure 1A (right panel), PANC-1 clones with high levels of brachyury (pBr cl-1 and cl-2) demonstrated a marked decrease in susceptibility to lysis by recombinant TRAIL, compared with PANC-1 clones that express very low/undetectable levels of brachyury (pBr cl-3 and cl-4). Similar results were obtained with single clonal populations of lung H460 cells with different levels of brachyury generated *via* the CRISPR/Cas9 methodology (Figure 1B, left panel). As shown in Figure 1B (right panel), H460 cells with high levels of brachyury (Hi) were lysed less efficiently than those with undetectable brachyury (Lo).

**Figure 2:**
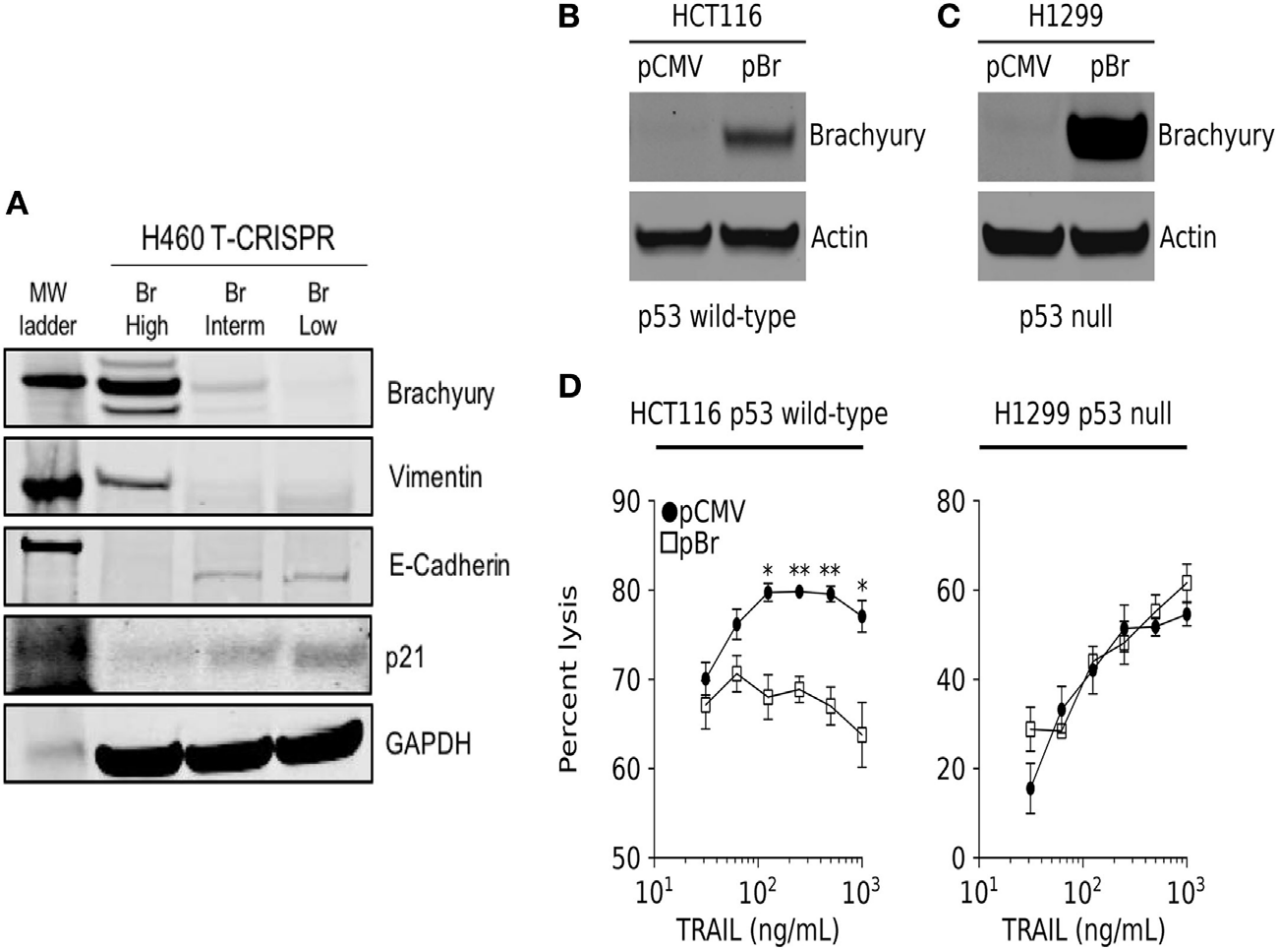
Brachyury drives resistance to TNF-related apoptosis-inducing ligand (TRAIL) in cells expressing a wild-type p53 gene. **(A)** Western blot analysis of brachyury, vimentin, E-cadherin, and p21 in H460 clones generated *via* the CRISPR/Cas9 system. **(B,C)** Western blot analysis of brachyury expression in HCT116 and H1229 cells, respectively, stably transfected with either a control or brachyury-encoding (pBr) plasmid. **(D)** The impact of brachyury expression on the sensitivity of HCT116 (left panel) and H1229 (right panel) to lysis by a range of TRAIL doses.

In a previous study, we have described the transcriptional repression of p21 as a mechanism responsible for the decreased susceptibility of brachyury-high carcinoma cells to chemotherapy and radiation. In accordance with those previous observations, clonal populations of lung H460 cells with various levels of brachyury (Figure 2A) demonstrated an inverse association between brachyury and p21 protein expression, whereby H460 cells with high expression of brachyury (Br-High) were characterized by high levels of mesenchymal vimentin, low levels of epithelial E-cadherin and very low levels of p21, while cells with lower brachyury levels (Br-Interm and Br-Low) exhibited reduced expression of vimentin, high expression of E-cadherin, and enhanced expression of the cell cycle regulator p21 (Figure 2A). Since expression of p21 is primarily regulated by p53-mediated response to DNA damage, we hypothesized that the p53 status of the cancer cells may impact the ability of brachyury to induce resistance to cytotoxic killing. To examine this possibility, brachyury was overexpressed in parallel experiments in the colon carcinoma line, HCT116, which carries wild-type p53, and the non-small cell lung carcinoma (NSCLC) line, H1299, which express a non-functional isoform of p53 (Figures 2B,C). These isogenic brachyury high (pBr) vs. low (pCMV) tumor cell pairs were then assayed for their susceptibility to lysis by recombinant TRAIL. As shown in Figure 2D, brachyury-associated reduction in TRAIL susceptibility was only observed in the p53 wild-type cell line, HCT116, while no effect of brachyury overexpression was observed in the p53 null H1299 cells.

### Reconstitution of p21 in Brachyury-High Cells Increases Sensitivity to Immune Cells

**Figure 3:**
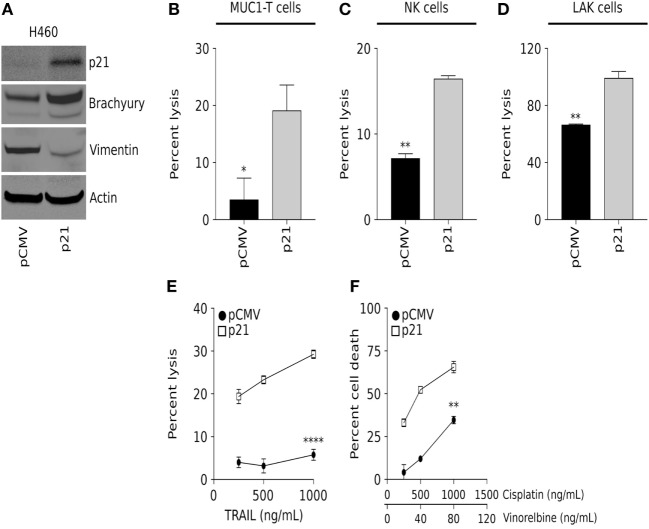
Overexpression of p21 in the brachyury-high H460 cell line increases sensitivity to lysis by both immune cells and chemotherapy. **(A)** Western blot analysis of p21, brachyury, and vimentin in H460 cells transfected to express either a control pCMV or a vector encoding p21 (p21). Sensitivity of H460-pCMV vs. H460-p21 cells to killing by **(B)** MUC-1-specific T cells (E:T ratio 30:1); **(C)** natural killer (NK) cells (E:T ratio 30:1); **(D)** lymphokine-activated killer (LAK) cells (E:T ratio 30:1); and indicated concentrations of **(E)** TNF-related apoptosis-inducing ligand (TRAIL) and **(F)** chemotherapy. NK and LAK lysis data presented is representative of data obtained using effector cells isolated from two different normal donors. TRAIL and chemotherapy data presented are representative of two independent experiments.

To further examine the role of p21 in brachyury-mediated resistance to immune-mediated killing of carcinoma cells, a non-clonal, heterogeneous population of parental H460 cells characterized by very high levels of brachyury expression and a mesenchymal phenotype, were transfected with an empty vector (pCMV) or a plasmid encoding human p21 under the control of the CMV promoter (Figure 3A). Using this isogenic pair of p21-low vs. p21-high cells, susceptibility to immune lysis was evaluated. As shown in Figures 3B–E, brachyury-high/p21-high cells (H460-p21) were efficiently lysed by MUC1-specific T cells, NK cells, and LAK cells and recombinant TRAIL, compared with low levels of lysis observed with brachyury-high/p21-low cells (H460-pCMV). This increased sensitivity to cell death was not restricted to immune-mediated lysis, as the H460-p21 cells were also more sensitive to killing by a combination of cisplatin and vinorelbine chemotherapy (Figure 3F) than tumor cells with low levels of p21 (H460-pCMV). Interestingly, overexpression of p21 in these cells was associated with a reduction of vimentin expression (Figure 3A), an observation in agreement with several previous reports on the ability of p21 to inhibit the epithelial–mesenchymal switch in tumor cells (25).

### Reconstitution of p21 Stabilized the CDK1 Protein in Brachyury-High Tumor Cells

**Figure 4:**
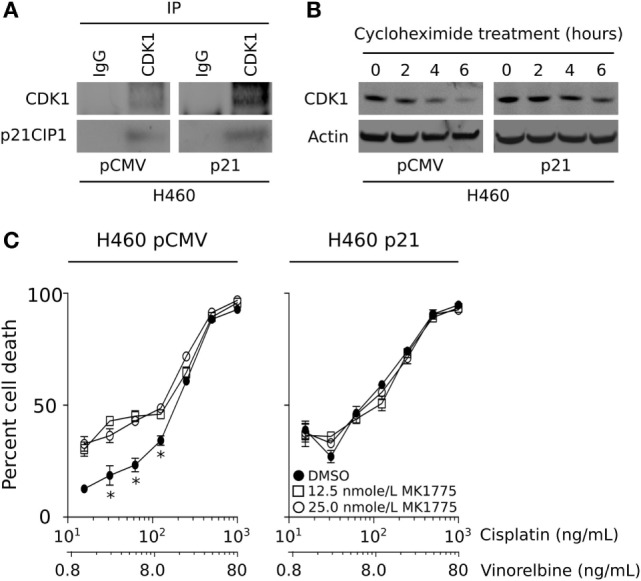
CDK1 protein is stabilized by p21, and WEE1 inhibition improves the sensitivity of brachyury-high/p21-low carcinoma cells. **(A)** Co-immunoprecipitation of CDK1 and p21 in lysates from H460 cells expressing either a control pCMV or a vector encoding p21. **(B)** CDK1 protein stability was evaluated in the H460 tumor cell pair following the addition of 100 µg/mL cycloheximide for the indicated time points. **(C)** Impact of MK-1775 pre-treatment (at indicated doses) on the sensitivity of H460 cells expressing a control pCMV, but not a p21 vector, to killing by chemotherapy. The data depicting the stability of CDK1, and chemotherapy sensitivity are representative of two independent experiments.

In a previous study, we demonstrated that brachyury-mediated immune resistance associates with decreased levels of the cyclin-dependent kinase 1 (CDK1) protein, and that reconstitution of CDK1 levels could restore the tumor cells’ susceptibility to immune effector cells. The mechanism by which high levels of brachyury decrease the expression of CDK1 protein was previously identified as a reduction of CDK1 protein stability in brachyury-high tumor cells. To investigate a potential association between the loss of CDK1 protein and reduction of p21, here, we have conducted co-immunoprecipitation assays using an anti-CDK1 antibody to investigate whether these two proteins could be associated in tumor cells with high vs. low brachyury levels. As shown in Figure 4A, p21 co-immunoprecipitated with CDK1 in H460-p21 cells demonstrating that these proteins may form a molecular complex *in vivo*. Interestingly, evaluation of the stability of CDK1 in the brachyury-high H460 cells demonstrated that reconstitution of p21 in the H460-p21 cells could increase the stability of CDK1 over that observed in H460-pCMV cells (Figure 4B).

One potential therapeutic avenue for improving the lysis of brachyury-high tumor cells consists of tumor pretreatment with an inhibitor of the G2 checkpoint kinase, WEE1, which normally suppresses the activity of CDK1 *via* phosphorylation on Tyr-15. We have previously shown that treatment of brachyury-high tumor cells with the WEE1 inhibitor, MK-1775, is able to overcome tumor resistance to immune-mediated killing by decreasing WEE1 activity and increasing the level of functional CDK1. WEE1 inhibition is currently undergoing clinical evaluation in combination with various conventional chemotherapeutic treatment strategies, particularly in patients whose tumors lack a functional p53 and, therefore, are dependent on the activity of the G2 cell cycle checkpoint (26). Our observations, however, suggest that high levels of brachyury, which negatively regulates the transcription of CDKN1A, may impart a p53 null phenotype in tumors with a functional p53 protein. To examine this further, we evaluated the impact of MK-1775 treatment on the susceptibility of H460 cells expressing either low (pCMV) or high levels of p21. As shown in Figure 4C, WEE1 inhibition was only able to improve the sensitivity of brachyury-high/p21-low (H460-pCMV) cells in response to chemotherapy, while the same treatment had no impact on the susceptibility of H460 cells over-expressing p21 (H460-p21).

### WEE1 Inhibition Improves the Sensitivity of Brachyury High/p21-Low Carcinoma Cells

**Figure 5:**
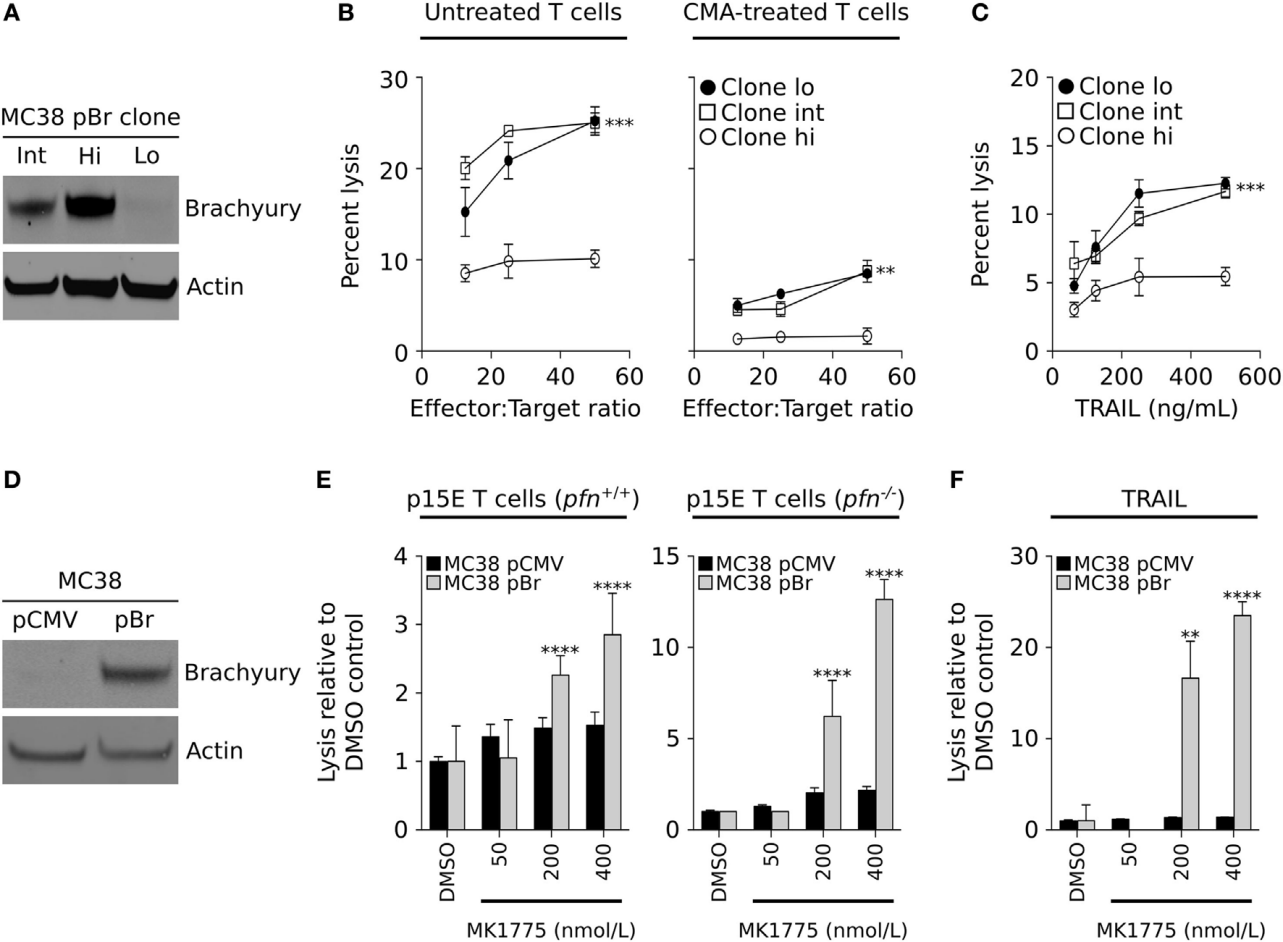
WEE1 inhibition improves the sensitivity of brachyury high/p21-low carcinoma cells. **(A)** Western blot analysis of brachyury expression in three clonally derived MC38 cell populations expressing low (Lo), intermediate (Int), or high (Hi) levels of the human brachyury transgene. **(B)** Sensitivity to lysis by p15E-specific cytotoxic CD8^+^ T cells, which have been left untreated (left panel) or were pre-treated with CMA to inhibit their ability to lyse targets in a perforin-dependent manner (right panel). **(C)** Sensitivity of MC38 clones to indicated doses of recombinant TNF-related apoptosis-inducing ligand (TRAIL). **(D)** Western blot analysis of brachyury expression in the MC38 pCMV and pBr isogenic cell pair. **(E)** Lysis of the MC38 tumor pair following WEE1 inhibition with indicated doses of MK-1775 by p15E-specific T cells (E:T ratio 50:1) expanded from either wild-type mice (left panel) or perforin-deficient animals (right panel), or **(F)** TRAIL (125 ng/mL). The data depicting the lysis of the MC38 pCMV and pBr cell lines are representative of two independent experiments.

To evaluate our results in a murine carcinoma model, the colon carcinoma cell line MC38 was stable transfected with a plasmid vector encoding the full-length brachyury protein, followed by single-cell cloning and generation of three cell lines, MC38-Lo, -Int, and -Hi, with either low, intermediate, or high levels of brachyury, respectively (Figure 5A). The sensitivity of these cells to lysis by CD8^+^ T cells specific for an epitope (p15E) of the endogenous retroviral env protein GP70 was evaluated. As shown in Figure 5B (left panel), MC38 cells expressing high levels of brachyury were poorly lysed in comparison with MC38 cells expressing either low or intermediate levels of brachyury. Immune effector cells can lyse targets either by using the FAS/TRAIL-mediated induction of the extrinsic caspase-dependent apoptotic pathway, or *via* the perforin-dependent actions of granzymes that lyse target cells in a caspase-dependent and/or independent fashion. Pretreatment of the p15E-specific CD8^+^ T cells with CMA was used to abrogate the activity of the perforin/granzyme lytic pathway. As shown in Figure 5B (right panel), CMA completely abolished the T cells’ ability to lyse MC38 cells with high levels of brachyury while the clones expressing intermediate and low levels were still lysed. The tumor resistant phenotype induced by high levels of brachyury was also observed when recombinant TRAIL was used to directly trigger the extrinsic apoptotic pathway (Figure 5C). The impact of WEE1 inhibition on the restoration of lysis of brachyury-high cells was also evaluated with MC38 cells transfected with an empty vector (pCMV) or a vector encoding the full-length brachyury protein (pBr, Figure 5D). As immune effector cells, murine p15E-specific T cells were used, generated either in wild-type (*pfn^+/+^*) or perforin deficient (*pfn^−/−^*) mice. As shown in Figure 5E, MK-1775 treatment enhanced T-cell mediated lysis of MC38-pBr cells, an effect that was exacerbated when T cells originating from perforin-deficient animals were used. In contrast, brachyury-low MC38-pCMV targets were not affected by treatment with MK-1775. Similar results were obtained when utilizing recombinant TRAIL (Figure 5F). While MC38-pCMV were highly susceptible to the effect of TRAIL and were not affected by MK-1775 treatment, MC38-pBr cells appeared resistant to the lytic activity of TRAIL and treatment with MK-1775 was able to significantly enhance their lysis in a dose-dependent way.

### Inverse Association Between Brachyury and p21 in Tumor Tissues

**Figure 6:**
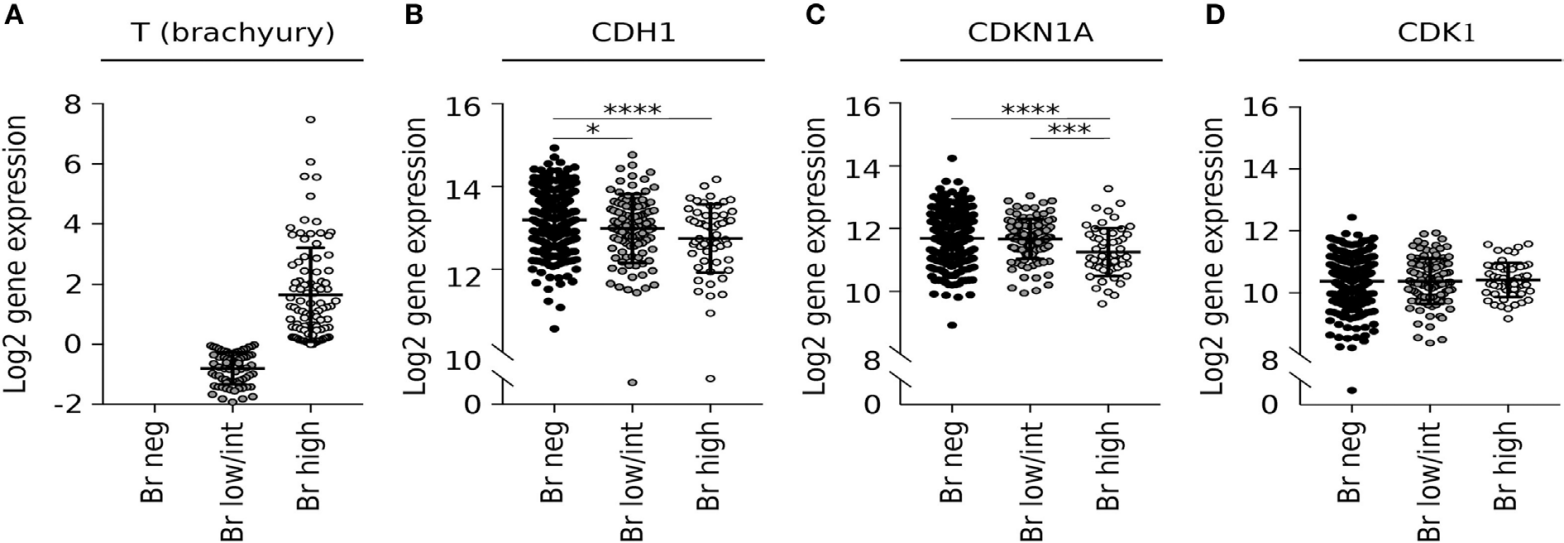
Brachyury mRNA correlates with low expression of CDKN1A in lung squamous cell carcinoma samples. Analysis of expression of **(A)** brachyury (*T*), **(B)** E-cadherin (*CDH1*), **(C)** p21 (*CDKN1A*), and **(D)** CDK1 transcripts in the LUSC The Cancer Genome Atlas dataset tabulated according to the level of brachyury mRNA.

Our preclinical observation on the existence of a negative correlation between brachyury and p21 expression in tumor cells was then corroborated *via* analysis of the lung squamous cell carcinoma dataset from (TCGA) for levels of mRNA encoding brachyury (*T*) and p21 (*CDKN1A*). When samples were separated into three subgroups based upon the level of brachyury expression (Figure 6A), it was observed that tumors expressing the highest levels of *T* mRNA had the lowest levels of CDH1 mRNA (encoding epithelial E-cadherin) and *CDKN1A* mRNA (encoding p21), as compared with the brachyury negative and low/intermediate groups (Figures 6B,C). Unlike with *CDKN1A*, no differences in *CDK1* mRNA levels were observed in relation to the levels of *T* mRNA (Figure 6D), which agrees with our previous observations that high levels of brachyury induce a reduction in CDK1 protein stability rather than a reduced transcriptional activity.

## Discussion

The phenomenon of cancer phenotypic plasticity, manifested as a modulation of tumor phenotype between the epithelial and mesenchymal-like states, is now considered an important mechanism in tumor progression (27, 28). Perhaps one of the most relevant consequences of this phenotypic modulation is the resulting acquisition of tumor cell resistance to a range of cell death-inducing signals, including those initiated by chemotherapy, radiation, some small molecule-targeted therapies and, as more recently shown, resistance to immune-mediated cytotoxicity. In the current study, the mechanism of immune resistance of carcinoma cells bearing brachyury-driven mesenchymal features was elucidated. The data demonstrate the loss of the cell cycle regulatory protein p21 during mesenchymalization is responsible for the decreased susceptibility of brachyury-high tumor cells to immune-mediated attack.

Several reports have now demonstrated a link between the acquisition of mesenchymal features by carcinoma cells and escape from immune cytotoxicity (17, 29–33). For example, the overexpression of the transcription factor Snail, a known mediator of the phenomenon of epithelial–mesenchymal transition, has been shown to impair apoptosis in response to TNF-α by decreasing the activity of initiator caspase-8 and effector caspase-3 (34). Akalay and colleagues also demonstrated that Snail overexpression leads to tumor decreased susceptibility to T-cell mediated lysis *via* the activation of autophagy (32). In the case of brachyury-driven mesenchymalization, we have previously reported that brachyury-high carcinoma cells are less susceptible to the cytotoxic activity of immune effector cells, including antigen-specific cytotoxic T cells, NK, or LAK cells, compared with brachyury-low cancer cells (17, 33). In the present work, clonal populations of brachyury high/mesenchymal cells vs. brachyury low/epithelial cells were compared side-by-side. The results of these experiments, as shown in Figures 1A,B and 5B, consistently showed tumor cells with a mesenchymal phenotype being less susceptible to immune attack than the epithelial counterparts. Previously, we also showed that expression of brachyury in carcinoma cells does not affect the levels of MHC-class I or beta-2 microglobulin expression. Moreover, brachyury does not reduce the expression of various components of the antigen processing and presentation machinery, including TAP1, TAP2, tapasin, LMP2, and LMP7 (17), thus ruling out a defect in antigen presentation as the cause of reduced sensitivity to immune attack. Rather, we showed that brachyury induced a blockade of apoptosis even in the presence of normal levels of activated caspases, which was associated with the defective phosphorylation and cleavage of the nuclear lamins following triggering (35, 36). This defect was due to the loss of the protein kinase CDK1 in brachyury-high tumor cells, although the mechanism of CDK1 protein destabilization was not understood at the time.

The p21 protein, initially identified as an inhibitor of cyclin-dependent kinases (37), has been described as a central player in multiple cell pathways, including (a) a direct target of p53 and a mediator of p53-dependent cell cycle arrest during DNA damage (38); (b) a protein involved in senescence and aging (39); and (c) a regulator of reprogramming in pluripotent stem cells (40). In certain conditions, p21 has also been shown to promote cell proliferation and oncogenicity. For example, p21 has been shown to act as a major adaptor protein that assembles cyclin D1/CDK4 complexes, targeting them into the nucleus and favoring cyclin D1-associated kinase activity (41). Thus, the p21 protein could exhibit both tumor suppressor and oncogenic properties, depending on the cellular context (42). In previous studies, the impact of brachyury overexpression in the proliferation of human carcinoma cells has been described. As shown with other regulators of the epithelial–mesenchymal phenomenon, expression of brachyury in carcinoma cells associates with a significant reduction in cell proliferation, whereby brachyury expression inversely correlates with the expression of phosphorylated Rb, Cyclin D1, and p21. In this context, our laboratory showed that forced upregulation of p21 was sufficient to reconstitute cell proliferation in high brachyury cells (23). Interestingly, there have been previous reports describing the involvement of p21 in tumor phenotypic plasticity. Liu et al., for example, demonstrated that deletion of p21 in transgenic mice not only accelerates mammary oncogenesis induced by MMTV-Ras and MMTV-c-Myc but also induces the acquisition of mesenchymal and stemness features in mammary tumors, *in vivo* (43). Similar observations with mammary carcinoma cells demonstrated that silencing of p21 and PUMA, a target of p53, leads to loss of E-cadherin and increased expression of markers of the epithelial–mesenchymal phenotypic switch (44). In subsequent studies, p21 has been shown to modulate the expression of various miRNAs, including miR-200 and the miR-183 cluster, which in turn regulate the expression of genes involved in the epithelial–mesenchymal switch. In various model systems, silencing of p21 increased mesenchymal features, including vimentin, fibronectin, N-cadherin, Slug, and Zeb1 expression, and increased tumor cell migration and invasiveness (25). The present report demonstrates that reconstitution of p21 in brachyury-high tumor cells markedly improves tumor cell lysis mediated by antigen-specific T cells, NK and LAK cells, TRAIL, and chemotherapy. In addition, reconstitution of p21 in tumor cells with high levels of brachyury is shown here to increase the stability of the CDK1 protein, leading to higher levels of CDK1 expression and the anticipated increase in tumor susceptibility to lysis. Our observations that the loss of p21 in brachyury-high carcinoma cells drives the acquisition of resistance to cytotoxic killing are in line with the concept that loss of this protein would also promote the acquisition of a more mesenchymal and resistant tumor status. It remains to be investigated, however, whether the expression of cytokines, chemokines, or growth factors secreted by immune effector T cells and NK cells co-cultured with tumor cells with various levels of p21 are different. These studies will help understand if the mesenchymalization phenomenon could potentially alter the recruitment, expansion, and level of activation of various populations of immune cells *via* secretion of a different set of soluble factors.

The WEE1 kinase regulates the G2 checkpoint in response to DNA damage, where WEE1 prevents entry into mitosis by mediating the inhibitory phosphorylation of CDK1. Previous studies conducted with WEE1 inhibition have shown that the effect of this inhibitor is mainly observed in the context of p53 mutant tumors, where the G1 checkpoint is lost and cells solely rely on the G2 checkpoint for cell cycle arrest following exposure to genotoxic agents (45–47). Interestingly, while brachyury overexpression in p53 wild-type cells resulted in decreased susceptibility to apoptosis triggered by TRAIL, no effect was observed when brachyury was overexpressed in tumor cells deficient for p53 (p53 null). These results suggested that high levels of brachyury, which negatively regulates the transcription of p21, may impart a p53 null phenotype in tumors with a functional p53 protein. In agreement with those results, inhibition of WEE1 kinase *via* treatment with MK-1775 improved the lysis of brachyury-high tumor cells only in the absence of p21, while the effect was lost when p21 was forcibly overexpressed in the presence of high brachyury.

The results of this study reinforce the concept that acquisition of mesenchymal features by carcinoma cells is accompanied by the acquisition of resistance mechanisms that allow tumor cells to evade not only cytotoxic therapies but also immune-mediated attack. This study also situates p21 as a central player in many of the aspects of the brachyury biology, including control of cell cycle, resistance to apoptosis induced by chemotherapy and, more importantly, resistance to immune-mediated lysis.

Several vaccine platforms targeting brachyury have now been developed and are undergoing clinical evaluation (14–16), based on the hypothesis that immunization against a driver of mesenchymalization could generate a T-cell response that would eradicate the population of cancer cells ultimately responsible for metastasis and relapse. We demonstrate here that the defects in tumor cell death described in association with very high levels of brachyury in tumor cells could be alleviated *via* the use of a WEE1 inhibitor, which is currently undergoing clinical testing (26). Our data demonstrate the potential usefulness of this inhibitor for improvement of immune resistance of carcinoma cells with mesenchymal features and high levels of brachyury expression. While most phase II clinical trials of WEE1 inhibition in combination with chemotherapy are conducted in patients with p53-defective tumors, we expect that our studies will provide rationale for the use of WEE1 inhibition in tumors regardless of p53 status, in combination with immunotherapeutic approaches against the transcription factor brachyury.

## Ethics Statement

Peripheral blood used in this study was obtained from healthy human donors recruited at the NIH Blood Bank (Bethesda, MD, USA), protocol number NCT00001846, under the appropriate NIH Institutional Review Board approval and informed consent. All animal studies were carried out in accordance with the guidelines of the Association for Assessment and Accreditation of Laboratory Animal Care (AAALAC). Experimental studies were carried out under approval of the NIH Intramural Animal Care and Use Committee.

## Author Contributions

DH designed and conducted experiments, analyzed data, and wrote the manuscript; KM conducted experiments and analyzed data; CP designed experiments, analyzed data, supervised the study, and wrote the manuscript.

## Conflict of Interest Statement

The authors declare that the research was conducted in the absence of any commercial or financial relationships that could be construed as a potential conflict of interest.

## References

[1] Fernando RI Litzinger M Trono P Hamilton DH Schlom J Palena C. The T-box transcription factor Brachyury promotes epithelial-mesenchymal transition in human tumor cells. J Clin Invest (2010) 120(2):533–44. 10.1172/JCI38379 20071775 PMC2810072

[2] Imajyo I Sugiura T Kobayashi Y Shimoda M Ishii K Akimoto N T-box transcription factor brachyury expression is correlated with epithelial-mesenchymal transition and lymph node metastasis in oral squamous cell carcinoma. Int J Oncol (2012) 41(6):1985–95. 10.3892/ijo.2012.1673 23076115 PMC3583627

[3] Du R Wu S Lv X Fang H Wu S Kang J. Overexpression of brachyury contributes to tumor metastasis by inducing epithelial-mesenchymal transition in hepatocellular carcinoma. J Exp Clin Cancer Res (2014) 33:105. 10.1186/s13046-014-0105-6 25499255 PMC4279691

[4] Pinto F Pertega-Gomes N Pereira MS Vizcaino JR Monteiro P Henrique RM T-box transcription factor Brachyury is associated with prostate cancer progression and aggressiveness. Clin Cancer Res (2014) 20(18):4949–61. 10.1158/1078-0432.CCR-14-0421 25009296

[5] Xu K Liu B Liu Y. Impact of brachyury on epithelial-mesenchymal transitions and chemosensitivity in non-small cell lung cancer. Mol Med Rep (2015) 12(1):995–1001. 10.3892/mmr.2015.3348 25683840 PMC4438917

[6] Roselli M Fernando RI Guadagni F Spila A Alessandroni J Palmirotta R Brachyury, a driver of the epithelial-mesenchymal transition, is overexpressed in human lung tumors: an opportunity for novel interventions against lung cancer. Clin Cancer Res (2012) 18(14):3868–79. 10.1158/1078-0432.CCR-11-3211 22611028 PMC3472640

[7] Miettinen M Wang Z Lasota J Heery C Schlom J Palena C. Nuclear brachyury expression is consistent in chordoma, common in germ cell tumors and small cell carcinomas, and rare in other carcinomas and sarcomas: an immunohistochemical study of 5229 cases. Am J Surg Pathol (2015) 39(10):1305–12. 10.1097/PAS.0000000000000462 26099010 PMC4567944

[8] Palena C Roselli M Litzinger MT Ferroni P Costarelli L Spila A Overexpression of the EMT driver brachyury in breast carcinomas: association with poor prognosis. J Natl Cancer Inst (2014) 106(5):dju054. 10.1093/jnci/dju054 24815864 PMC4568990

[9] Hamilton DH Roselli M Ferroni P Costarelli L Cavaliere F Taffuri M Brachyury, a vaccine target, is overexpressed in triple-negative breast cancer. Endocr Relat Cancer (2016) 23(10):783–96. 10.1530/ERC-16-0037 27580659 PMC5010091

[10] Kilic N Feldhaus S Kilic E Tennstedt P Wicklein D Wasielewski R Brachyury expression predicts poor prognosis at early stages of colorectal cancer. Eur J Cancer (2011) 47(7):1080–5. 10.1016/j.ejca.2010.11.015 21220197

[11] Jezkova J Williams JS Pinto F Sammut SJ Williams GT Gollins S Brachyury identifies a class of enteroendocrine cells in normal human intestinal crypts and colorectal cancer. Oncotarget (2016) 7(10):11478–86. 10.18632/oncotarget.7202 26862851 PMC4905487

[12] Haro A Yano T Kohno M Yoshida T Koga T Okamoto T Expression of Brachyury gene is a significant prognostic factor for primary lung carcinoma. Ann Surg Oncol (2013) 20(Suppl 3):S509–16. 10.1245/s10434-013-2914-9 23456319

[13] Pinto F Pertega-Gomes N Vizcaino JR Andrade RP Carcano FM Reis RM. Brachyury as a potential modulator of androgen receptor activity and a key player in therapy resistance in prostate cancer. Oncotarget (2016) 7(20):28891–902. 10.18632/oncotarget.8499 27049720 PMC5045364

[14] Gabitzsch ES Tsang KY Palena C David JM Fantini M Kwilas A The generation and analyses of a novel combination of recombinant adenovirus vaccines targeting three tumor antigens as an immunotherapeutic. Oncotarget (2015) 6(31):31344–59. 10.18632/oncotarget.5181 26374823 PMC4741610

[15] Heery CR Singh BH Rauckhorst M Marte JL Donahue RN Grenga I Phase I trial of a yeast-based therapeutic cancer vaccine (GI-6301) targeting the transcription factor brachyury. Cancer Immunol Res (2015) 3(11):1248–56. 10.1158/2326-6066.CIR-15-0119 26130065 PMC4636967

[16] Heery CR Palena C McMahon S Donahue RN Lepone LM Grenga I Phase I study of a poxviral TRICOM-based vaccine directed against the transcription factor brachyury. Clin Cancer Res (2017) 23(22):6833–45. 10.1158/1078-0432.CCR-17-1087 28855356 PMC5690815

[17] Hamilton DH Huang B Fernando RI Tsang KY Palena C. WEE1 inhibition alleviates resistance to immune attack of tumor cells undergoing epithelial-mesenchymal transition. Cancer Res (2014) 74(9):2510–9. 10.1158/0008-5472.CAN-13-1894 24626094 PMC4011139

[18] Terry S Savagner P Ortiz-Cuaran S Mahjoubi L Saintigny P Thiery JP New insights into the role of EMT in tumor immune escape. Mol Oncol (2017) 11(7):824–46. 10.1002/1878-0261.12093 28614624 PMC5496499

[19] Fox BA Spiess PJ Kasid A Puri R Mule JJ Weber JS In vitro and in vivo antitumor properties of a T-cell clone generated from murine tumor-infiltrating lymphocytes. J Biol Response Mod (1990) 9(5):499–511. 2174966

[20] Tsang KY Palena C Gulley J Arlen P Schlom J. A human cytotoxic T-lymphocyte epitope and its agonist epitope from the nonvariable number of tandem repeat sequence of MUC-1. Clin Cancer Res (2004) 10(6):2139–49. 10.1158/1078-0432.CCR-1011-03 15041735

[21] Palena C Polev DE Tsang KY Fernando RI Litzinger M Krukovskaya LL The human T-box mesodermal transcription factor brachyury is a candidate target for T-cell-mediated cancer immunotherapy. Clin Cancer Res (2007) 13(8):2471–8. 10.1158/1078-0432.CCR-06-2353 17438107

[22] Hamilton DH Fernando RI Schlom J Palena C. Aberrant expression of the embryonic transcription factor brachyury in human tumors detected with a novel rabbit monoclonal antibody. Oncotarget (2015) 6(7):4853–62. 10.18632/oncotarget.3086 25605015 PMC4467120

[23] Huang B Cohen JR Fernando RI Hamilton DH Litzinger MT Hodge JW The embryonic transcription factor brachyury blocks cell cycle progression and mediates tumor resistance to conventional antitumor therapies. Cell Death Dis (2013) 4:e682. 10.1038/cddis.2013.208 23788039 PMC3702290

[24] Cancer Genome Atlas Network. Comprehensive molecular portraits of human breast tumours. Nature (2012) 490(7418):61–70. 10.1038/nature11412 23000897 PMC3465532

[25] Li XL Hara T Choi Y Subramanian M Francis P Bilke S A p21-ZEB1 complex inhibits epithelial-mesenchymal transition through the microRNA 183-96-182 cluster. Mol Cell Biol (2014) 34(3):533–50. 10.1128/MCB.01043-13 24277930 PMC3911499

[26] Do K Wilsker D Ji J Zlott J Freshwater T Kinders RJ Phase I study of single-agent AZD1775 (MK-1775), a Wee1 kinase inhibitor, in patients with refractory solid tumors. J Clin Oncol (2015) 33(30):3409–15. 10.1200/JCO.2014.60.4009 25964244 PMC4606059

[27] Savagner P. Epithelial-mesenchymal transitions: from cell plasticity to concept elasticity. Curr Top Dev Biol (2015) 112:273–300. 10.1016/bs.ctdb.2014.11.021 25733143

[28] Nieto MA Huang RY Jackson RA Thiery JP. Emt: 2016. Cell (2016) 166(1):21–45. 10.1016/j.cell.2016.06.028 27368099

[29] Kudo-Saito C Shirako H Takeuchi T Kawakami Y. Cancer metastasis is accelerated through immunosuppression during Snail-induced EMT of cancer cells. Cancer Cell (2009) 15(3):195–206. 10.1016/j.ccr.2009.01.023 19249678

[30] Santisteban M Reiman JM Asiedu MK Behrens MD Nassar A Kalli KR Immune-induced epithelial to mesenchymal transition in vivo generates breast cancer stem cells. Cancer Res (2009) 69(7):2887–95. 10.1158/0008-5472.CAN-08-3343 19276366 PMC2664865

[31] Reiman JM Knutson KL Radisky DC. Immune promotion of epithelial-mesenchymal transition and generation of breast cancer stem cells. Cancer Res (2010) 70(8):3005–8. 10.1158/0008-5472.CAN-09-4041 20395197 PMC2856111

[32] Akalay I Janji B Hasmim M Noman MZ Andre F De Cremoux P Epithelial-to-mesenchymal transition and autophagy induction in breast carcinoma promote escape from T-cell-mediated lysis. Cancer Res (2013) 73(8):2418–27. 10.1158/0008-5472.CAN-12-2432 23436798

[33] David JM Hamilton DH Palena C. MUC1 upregulation promotes immune resistance in tumor cells undergoing brachyury-mediated epithelial-mesenchymal transition. Oncoimmunology (2016) 5(4):e1117738. 10.1080/2162402X.2015.1117738 27141403 PMC4839328

[34] Vega S Morales AV Ocana OH Valdes F Fabregat I Nieto MA. Snail blocks the cell cycle and confers resistance to cell death. Genes Dev (2004) 18(10):1131–43. 10.1101/gad.294104 15155580 PMC415638

[35] Ottaviano Y Gerace L. Phosphorylation of the nuclear lamins during interphase and mitosis. J Biol Chem (1985) 260(1):624–32. 3965465

[36] Rao L Perez D White E. Lamin proteolysis facilitates nuclear events during apoptosis. J Cell Biol (1996) 135(6 Pt 1):1441–55. 10.1083/jcb.135.6.1441 8978814 PMC2133948

[37] Harper JW Adami GR Wei N Keyomarsi K Elledge SJ. The p21 Cdk-interacting protein Cip1 is a potent inhibitor of G1 cyclin-dependent kinases. Cell (1993) 75(4):805–16. 10.1016/0092-8674(93)90499-G 8242751

[38] Waldman T Kinzler KW Vogelstein B. p21 is necessary for the p53-mediated G1 arrest in human cancer cells. Cancer Res (1995) 55(22):5187–90. 7585571

[39] Brown JP Wei W Sedivy JM. Bypass of senescence after disruption of p21CIP1/WAF1 gene in normal diploid human fibroblasts. Science (1997) 277(5327):831–4. 10.1126/science.277.5327.831 9242615

[40] Kawamura T Suzuki J Wang YV Menendez S Morera LB Raya A Linking the p53 tumour suppressor pathway to somatic cell reprogramming. Nature (2009) 460(7259):1140–4. 10.1038/nature08311 19668186 PMC2735889

[41] LaBaer J Garrett MD Stevenson LF Slingerland JM Sandhu C Chou HS New functional activities for the p21 family of CDK inhibitors. Genes Dev (1997) 11(7):847–62. 10.1101/gad.11.7.847 9106657

[42] Roninson IB. Oncogenic functions of tumour suppressor p21(Waf1/Cip1/Sdi1): association with cell senescence and tumour-promoting activities of stromal fibroblasts. Cancer Lett (2002) 179(1):1–14. 10.1016/S0304-3835(01)00847-3 11880176

[43] Liu M Casimiro MC Wang C Shirley LA Jiao X Katiyar S p21CIP1 attenuates Ras- and c-Myc-dependent breast tumor epithelial mesenchymal transition and cancer stem cell-like gene expression in vivo. Proc Natl Acad Sci U S A (2009) 106(45):19035–9. 10.1073/pnas.0910009106 19858489 PMC2776463

[44] Zhang Y Yan W Jung YS Chen X. PUMA cooperates with p21 to regulate mammary epithelial morphogenesis and epithelial-to-mesenchymal transition. PLoS One (2013) 8(6):e66464. 10.1371/journal.pone.0066464 23805223 PMC3689819

[45] Hirai H Iwasawa Y Okada M Arai T Nishibata T Kobayashi M Small-molecule inhibition of Wee1 kinase by MK-1775 selectively sensitizes p53-deficient tumor cells to DNA-damaging agents. Mol Cancer Ther (2009) 8(11):2992–3000. 10.1158/1535-7163.MCT-09-0463 19887545

[46] Bridges KA Hirai H Buser CA Brooks C Liu H Buchholz TA MK-1775, a novel Wee1 kinase inhibitor, radiosensitizes p53-defective human tumor cells. Clin Cancer Res (2011) 17(17):5638–48. 10.1158/1078-0432.CCR-11-0650 21799033 PMC3167033

[47] Rajeshkumar NV De Oliveira E Ottenhof N Watters J Brooks D Demuth T MK-1775, a potent Wee1 inhibitor, synergizes with gemcitabine to achieve tumor regressions, selectively in p53-deficient pancreatic cancer xenografts. Clin Cancer Res (2011) 17(9):2799–806. 10.1158/1078-0432.CCR-10-2580 21389100 PMC3307341

